# Acute stress induction downregulates RHOX-5 gene expression causing altered fertility indices in MALE Wistar rats

**DOI:** 10.5935/1518-0557.20250184

**Published:** 2026

**Authors:** Onyinye Cynthia Okeke, Olutayo Ifedayo Ajayi

**Affiliations:** 1 Department of Medical Physiology, School of Medicine and Pharmacy, College of Medicine and Health Sciences, University of Rwanda, Butare, Rwanda; 2 Department of Physiology, Basic Medical Sciences, University of Global Health Equity, Butaro, Rwanda

**Keywords:** stress, fertility hormones, infertility, Rhox-5 gene

## Abstract

**Objective:**

Stress has been implicated in the onset and development of various diseases and conditions including cardiovascular diseases, respiratory diseases, obesity and infertility. The effects of stress on fertility were studied to elucidate possible mechanisms of stress-induced infertility.

**Methods:**

Stress was induced using the flowerpot technique. Plasma concentration of fertility hormones, inflammatory and oxidative markers, sperm profile, histological studies and molecular studies were carried out in selected tissues from the study and control groups. Statistical analysis was carried out using the GraphPad Prism 8.0.1 version. Results were presented as Mean±SEM. The Student t-test was used to compare the means obtained; and p-values lower than 0.05 were considered statistically significant.

**Results:**

Plasma concentration of testosterone, percentage of progressively motile sperm cells, percentage of normal sperm cells and relative expression of Rhox-5 gene were significantly decreased, while the C-reactive protein concentration was significantly increased in the stressed group when compared with the control group.

**Conclusions:**

Stress reduces fertility through suppressed fertility hormone secretion and reduced genetic expression of the relevant genes, causing poor-quality spermatogenesis in male Wistar rats.

## INTRODUCTION

The prevalence of infertility amongst couples is becoming a cause for serious concern. Infertility has been linked with reproductive system disorders (Sabarre *et al*., 2013), age (Deyhoul *et al*., 2017), and hormonal disorders (Seth *et al*., 2013). Over the years, stress has been attributed to the onset or development of various diseases and conditions including cardiovascular diseases (Steptoe & Kivimäki, 2012), obesity (Scott *et al*., 2012), respiratory diseases (Aich *et al*., 2009), and infertility (Kalantaridou *et al*., 2010) amongst others. Stress has been linked to infertility in both males and females (Genazzani, 2005; Whirledge & Cidlowski, 2010; Dobson *et al*., 2012; Stárka & Dušková, 2015), with little knowledge of the exact mechanisms involved. A study by Andersen *et al*. (2005) revealed that stress causes alteration in levels of reproductive hormones. Likewise, Whirledge & Cidlowski (2010), reported that modifications on the hypothalamic-pituitary-adrenal (HPA) axis, with the resultant changes in circulating levels of glucocorticoids in response to stress, leading to an inhibition in reproduction.

The reproductive system in both males and females is controlled by hormonal secretions from the hypothalamic-pituitary-gonadal (HPG) axis (Meccariello *et al*., 2014), with the secretion of gonadotropin-releasing hormone (GnRH) at the level of the hypothalamus; gonadotropins at the pituitary gland and testosterone from the testis, each hormone acting to stimulate or suppress the secretion of another in a classical feedback manner.

Stressful conditions result in the activation of the hypothalamic-pituitary-adrenal axis (HPA) - a key component of the stress system (Chrousos, 2009), as such, its integrity and precise regulation are essential for adapting to stressors. Increased levels of glucocorticoid, a principal component of the HPA axis, promotes gluconeogenesis and mobilization of amino acids, actions required to maintain circulating levels of glucose to mount a stress response (Whirledge & Cidlowski, 2010). It has also been extensively documented that elevated levels of glucocorticoids affect gonadal function at multiple levels of the HPG axis (Maeda & Tsukamura, 2006; Whirledge & Cidlowski, 2010). Decreased testosterone production, sperm production, maturation, erectile dysfunction, or impotence have been observed with stress (Daly *et al*., 2005; Whirledge & Cidlowski, 2010).

Infertility has been reported to cause stress (Chen *et al*., 2004; Ilacqua *et al*., 2018; Rooney & Domar, 2018), but the question of whether stress causes infertility has not been sufficiently answered. Various methods have been experimentally used to induce stress such as deprivation paradigms (Andersen *et al*., 2005), electric foot shocks (Cakır *et al*., 2010), forced swimming, and the restraint method (Jameel *et al*., 2014).

Rhox-5 and Cox-2 genes, both expressed in the Sertoli cells and Leydig cells respectively, have been observed to play active roles in gametogenesis and steroidogenesis. It has been reported that male mice lacking the Rhox-5 gene are sub-fertile, exhibiting increased germ cell apoptosis and a defect in sperm mobility (Hu *et al*., 2007). Inhibition of the stAR protein by the Cox-2 gene has also been reported to lead to suppression of steroidogenesis (Wang *et al*., 2005).

Hence, this study was aimed at examining if stress causes infertility and investigate the possible mechanisms of how stress modulates the morphology and function of the male reproductive systems in Wistar rats.

## MATERIALS AND METHODS

### Setting of the study

The study was carried out in the Department of Physiology Laboratory, University of Benin, Edo state, Nigeria. The animals were handled in accordance with the Guiding Principles for Research as recommended by the Declaration of Helsinki and the Guiding Principles for the Care and Use of Laboratory Animals. Consent and approval from the Research and Ethics Committee of the above University and department were received before the study.

### Study design

Forty male and fifteen female Wistar rats (15 weeks old) weighing 250-280g were used for the study. They were housed in standard cages at room temperature, with 12 hours light/dark cycle. Before the study commenced, the animals were fed standard chow and water *ad libitum* and acclimatized for 2 weeks.

### Stress induction

The rats were stressed using a customized form of the flowerpot technique as described by Heinrichs & Koob (2006).

-a waterproof pool is prepared and flooded with water at room temperature,

-a cylindrical pedestal with a height of about 1cm above the water level in the pool was fixed to the center of the floor of the pool,

-the animal is placed on the pedestal and is brought out of the pool to eat and rest for 1 hour each day.

### Animal grouping

Forty male Wistar rats were sub-sectioned into 15 and 25 rats for sub-sections I and II, respectively. The animals in sub-section I were grouped into three (A, B, and C); with each group having 5 rats. The animals in groups A, B, and C were stressed using the flowerpot technique for 2, 3, and 5 days, respectively. The body weight of each rat was measured using an appropriate weighing balance, and blood samples were collected through the tail vein before and after stress induction for the evaluation of cortisol.

The rats in sub-section II were grouped into two. Five rats served as the control group, while 20 rats were stressed for 3 days and served as the stressed group. Fifteen animals from the stressed group were allowed to recover (recovery group) for 2, 4, and 6 weeks.

After stress induction and recovery, all the animals were sacrificed by cervical dislocation. Blood was drawn by direct cardiocentesis, centrifuged at 3500 RMP for 10 minutes and serum was collected for analysis of concentrations of follicle-stimulating hormone, luteinizing hormone, testosterone, anti-inflammatory, and antioxidant markers. Brain and testes were harvested for the pituitary gland and testicular histology. Testicular gene expression of Rhox-5 and Cox-2 were also investigated.

### Fertility test

Fifteen unstressed female Wistar were grouped into three and allowed to mate with male rats in a ratio of 2:1 as follows;

Group A - Unstressed males (UM) + unstressed females (UF) (Control group).
Group B - Stressed males (SM) + unstressed females (UF)
Group C - Recovered + unstressed females.

The mating was confirmed by obtaining a vaginal smear through pipetting normal saline into the vagina and the mixture obtained was placed on a glass slide, covered with a cover slip, and viewed under the microscope. When mating occurred, sperm cells were visible in the vagina smear. After mating confirmation, the outcome of pregnancy was investigated i.e., pregnancy success rate, litter weight, litter numbers, and litter survival.

### Hormone assays

The serum concentration of cortisol, follicle-stimulating hormone, luteinizing hormone, and testosterone were measured using an Enzyme-linked immunosorbent assay (ELISA) following the instructions on the manufacturer’s kit (Darwish, 2006). The Elisa kits used for the study were manufactured by Calbiotech Incorporation, United States.

### Sperm analysis

Sperm motility and morphology were studied using the methods described by Ibeh *et al*. (2020).

### Pituitary and testicular histology

The pituitary glands and testes were harvested and histological studies were done using standard methods of fixing, sectioning, and staining (Yang *et al*., 2006).

### Inflammatory markers assay

C-reactive protein was measured using an Enzyme-linked immunosorbent assay (ELISA) following the instructions on the manufacturer’s kit (Darwish, 2006). The Elisa kits were manufactured by the Calbiotech Incorporation; the United States were used for the study. Fibrinogen concentration was measured using the gravimetric assay method (Ajayi *et al*., 2015).

### Antioxidant assay

Catalase and superoxide dismutase activities were measured using spectrophotometry (Weydert & Cullen, 2010; Hadwan & Abed, 2015).

### Molecular assay

Specific gene expression analysis was done using the RT-PCR and gel electrophoresis technique (Mitchell & Iadarola, 2010). In the testes, Rhox-5 and Cox-2 genes were studied. The relative amount of cDNA (i.e., the intensities of the bands from agarose gel electrophoresis) was quantified through densitometry, using the ImageJ Software Version 5.0 and the gene expression was normalized with the GADPH gene as a housekeeping gene.

The sequence of primers used are as follows:

**Table:** 

S/N	Gene name	Forward primer sequence	Reverse primer sequence
1	Cox-2	GATTGACAGCCCACCAACTT	CGGGATGAACTCTCTCCTCA
2	Rhox-5	TCCAGTGGCGGGAGGAG	GGCACCCAGATGTTGCTCTA

The accession numbers for the primers used are as follows:

**Table:** 

S/N	Gene name	Accession number
1	Cox-2	NC_051348.1
2	Rhox-5	NM_022175.2

### Statistical Analysis

GraphPad Prism 8.0.1 (244) was used to analyze the data generated. Results are presented as Mean±SEM. Bar charts were plotted to show the results. Paired and unpaired student t-tests were used to compare the results in different groups and between various groups. P-values lower than 0.05 (*p*<0.05) were taken to be statistically significant.

## RESULTS

### Body weight and cortisol

**Figure 1:**
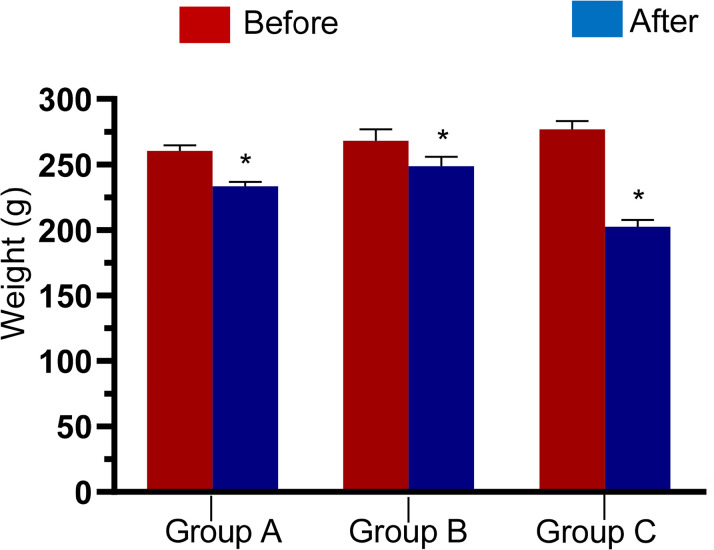
Weight of male Wistar rats before and after stress in Groups A, B, and C.

**Figure 2:**
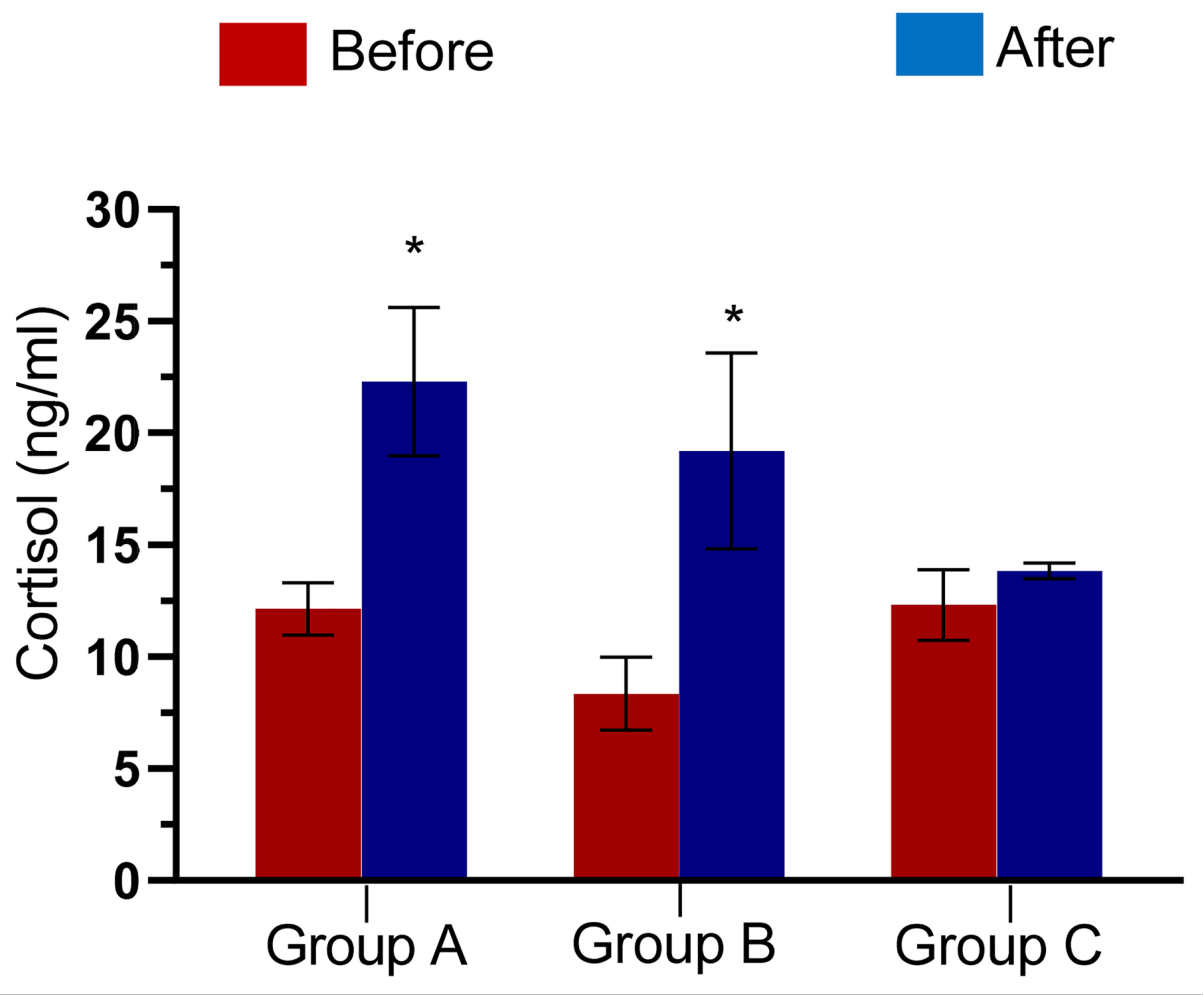
Serum Cortisol concentration of male Wistar rats before and after stress in Group A, B, and C. *p<0.05 is considered significant compared with the control.

Figure 1 shows a significant reduction (*p*<0.05) in the weight of male Wistar rats after stress for 2 days, 3 days, and 5 days. The serum concentration of cortisol was increased (*p*<0.05) after stress for 2 and 3 days but was not significantly different after 5 days of stress (Fig. 2).

### Hormones

**Figure 3:**
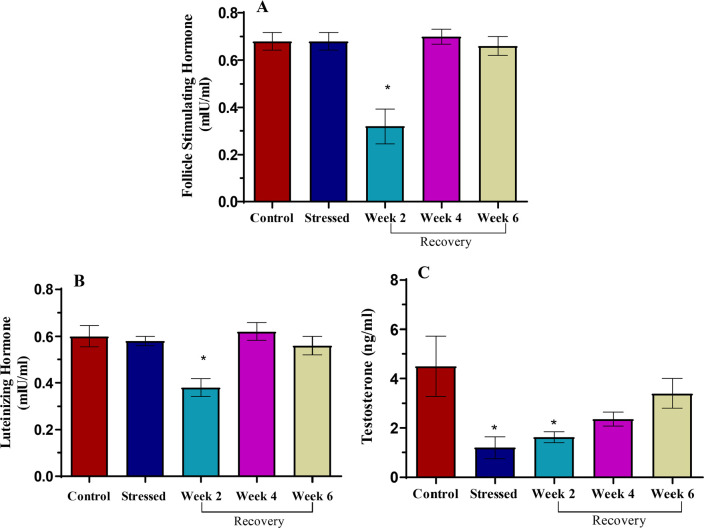
Serum follicle stimulating hormone, luteinizing hormone, and testosterone concentration in control, stressed and recovery groups of male Wistar rats. *p<0.05 is considered significant compared with the control.

No difference was observed in the concentration of follicle-stimulating hormone and luteinizing hormone (Fig. 3). There was a significant reduction (*p*<0.05) in the concentration of testosterone (Fig. 3), while there were significant reductions in follicle stimulating hormone, luteinizing hormone, and testosterone at week 2 of recovery (Fig. 3) (*p*<0.05, respectively).

### Sperm Profile

**Figure 4:**
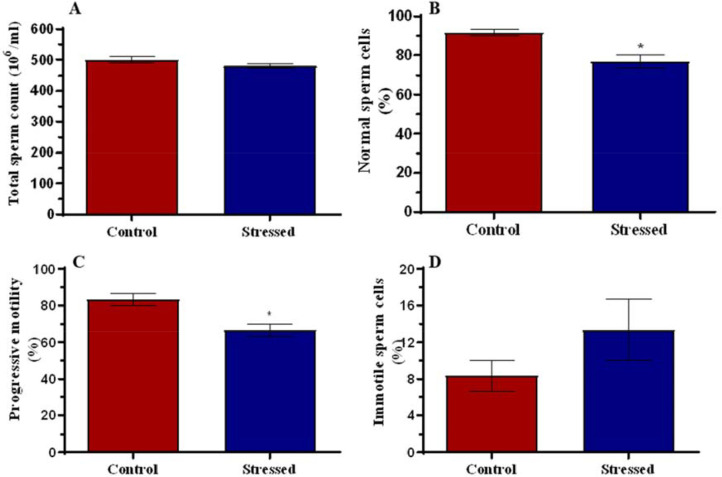
Total sperm count (A), percentage of normal sperm cells (B), progressive motility (C), and immotile sperm cells in control and stressed rats. *p<0.05 is considered significant compared with the control.

Figure 4A shows no difference in total sperm count. There was a significant reduction (*p*<0.05) in the percentage of normal sperm cells (Fig. 4B), and sperm cells with progressive motility (Fig. 4C). Percentage of immotile sperm cells was unchanged (Fig. 4D).

For sperm morphology, Table 1 shows the percentage of sperm cells in the control and stressed group which were headless, tailless, had big heads, short tails, double heads, head to head aggregation, tail to tail aggregation and body to body aggregation.

**Table 1:** The percentage abnormality of sperm cells in control and stressed Wistar rats.

Sperm abnormalities	Control	Stressed
Headless	5.00	6.67
Tailless	3.33	6.67
Big head	-	6.67
Short tail	-	5.00
Double head	-	1.67
Head-to-head	13.33	50.00
Tail to tail	20.00	23.33
Body to body	-	10.00

### Histology

**Figure 5:**
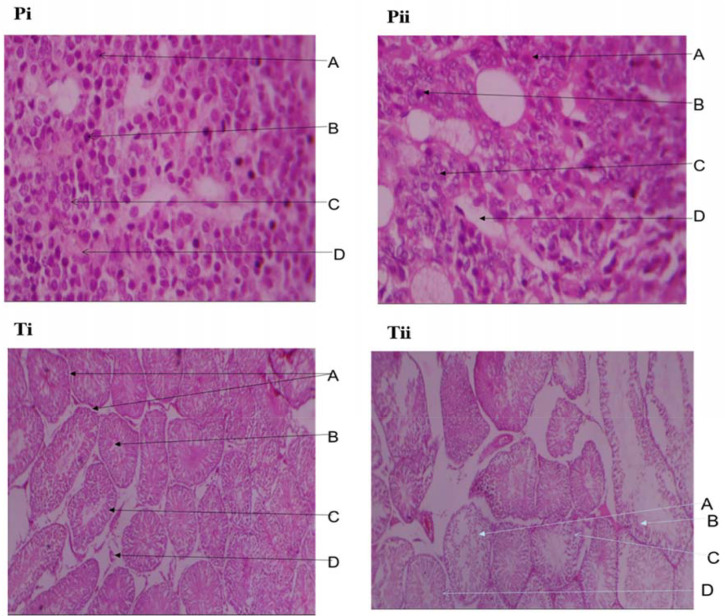
Photomicrograph of pituitary gland (Pi & ii) and testes (Ti & ii) of control and stressed male Wistar rats (H&E x 400).

Figure 5Pii shows that the pituitary gland of the stressed animals has decreased population and viability of acidophils (A) and basophils (B), compared with the control group (Fig. 5Pi); the chromophobes with inconspicuous nucleoli (C). The dilated and congested sinusoid (D) was observed in the stressed group when compared with the control. (Fig. 5Pi)

### Antioxidant markers

**Figure 6:**
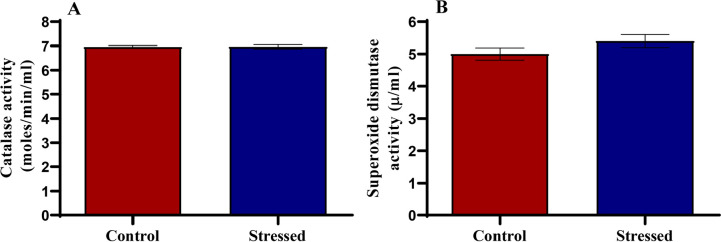
Catalase and superoxide dismutase activities in control and stressed male Wistar rats. *p<0.05 is considered significant compared with the control.

There was no difference in catalase activity (Fig. 6A) and superoxide dismutase activity (Fig. 6B).

### Anti-inflammatory markers

**Figure 7:**
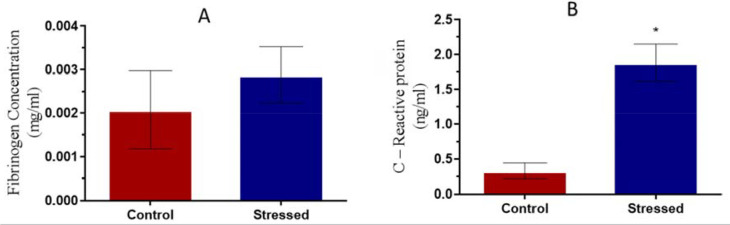
Fibrinogen and C-Reactive protein concentration in control and stressed male Wistar rats. *p<0.05 is considered significant compared with the control.

No difference was observed in fibrinogen concentration (Fig. 7A), but C-reactive protein was significantly (*p*<0.05) increased in the stressed group (Fig. 7B).

### Pregnancy outcome

Table 2 shows a reduced pregnancy success rate, a significant reduction in litter numbers, an unchanged litter survival rate, and litter weight in group B. The table also shows no difference in pregnancy success rate, litter numbers, litter survival rate, and litter weight in Group C.

**Table 2:** Pregnancy outcomes between Group A, Group B, and Group C Wistar rats.

	Group A	Group B	Group C
Pregnancy success rate (%)	100	40^*^	75
Litter numbers	6.40±0.51	2.40±1.47^*^	5.50±1.85
Litter survival rate (%)	84.38	83.33	81.81
Litter weight	0.22±0.004	0.21±0.01	0.21±0.005

* denotes statistical difference (*p*<0.05) when compared with control.

### Molecular Assay

**Figure 8:**
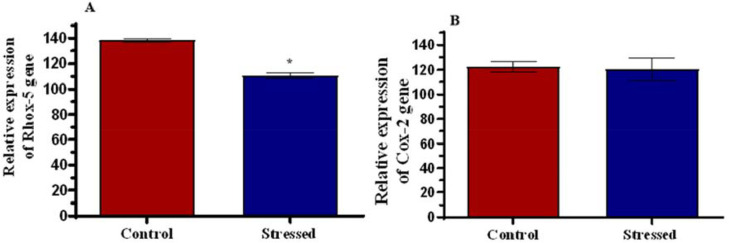
Relative expression of Rhox-5 gene (A) and Cox-2 gene (B) in control and stressed male Wistar rats. *p<0.05 is considered significant compared with the control.

There was a significant reduction in the relative expression of the Rhox-5 gene (Fig. 8A). Figure 8B shows the unchanged relative expression of the Cox-2 gene.

## DISCUSSION

This study employs the flowerpot technique of stress induction. This technique of stress induction works on various principles that have been observed to increase cortisol levels, hence inducing stress. They include rapid eye movement (REM) sleep deprivation (Andersen *et al*., 2005), restraint (Arakawa, 2020), food deprivation (Patchev & Patchev, 2006; Wunderink *et al*., 2012; Midwood *et al*., 2016), fear and anxiety (Adamec *et al*., 2005) and isolation (Heinrichs & Koob, 2006; Weiss *et al*., 2004).

Stress has been linked to infertility in both males and females (Genazzani, 2005; Whirledge & Cislowski, 2010; Dobson *et al*., 2012; Stárka & Dušková, 2015), with little knowledge of the exact mechanisms involved. A study by Andersen *et al*. (2005) revealed that stress causes alterations in levels of reproductive hormones. Likewise, Whirledge & Cidlowski (2010), observed that modification of the hypothalamic-pituitary-adrenal (HPA) axis, with the resultant changes in circulating levels of glucocorticoids in response to stress, leads to an inhibition of reproduction. This study was aimed at elucidating the possible mechanisms by which stress modulates the morphologies and functions of male Wistar rats, hence causing infertility. Various parameters measured in this study include body weight, the concentration of cortisol to establish stress, concentrations of fertility hormones, inflammatory and oxidative biomarkers.

The results showed a significantly decreased weight of the animals after the 2^nd^, 3^rd^, and also 5^th^ day of stress induction using the flowerpot technique. Acute stress has been associated with feeding suppression (anorexigenic effects) and reduced body weight gain (Halataei *et al*., 2011; Ranjbaran *et al*., 2013; Bagheri Nikoo *et al*., 2014; Rabasa & Dickson, 2016). Furthermore, the decrease in weight observed in this study could be attributed to the poor feeding of the animals since they were only fed for 1 hour daily, unlike before the experiment when they were fed ad libitum. Excessive gluconeogenesis and glycogenolysis characterize the neuroendocrine response to stress, leading to stress hyperglycemia (Dungan *et al*., 2009). During fasting, there is reduced glucose uptake to be stored in tissues, and there is increased glycogenolysis and gluconeogenesis to enrich the blood with glucose to sustain the tissues (Izumida *et al*., 2013), leading to a decrease in the body weight of the animal.

Serum cortisol concentrations were significantly increased in the groups stressed for 2 and 3 days using the flowerpot technique, supporting the observations of Cakır *et al*. (2010), who induced stress using electric foot shocks, and Jameel *et al*. (2014) who induced stress using forced swim and restraint methods of stress induction. Increased concentration of cortisol from the adrenal gland is normally prompted by an adrenocorticotropic hormone from the anterior pituitary glands; whose secretion is triggered by the release of Corticotropin-releasing hormone from the hypothalamus in response to stress (Gallo-Payet *et al*., 2017; Vaeroy *et al*., 2019). The results also show that for the animals stressed for 5 days, the serum concentration of cortisol remained statistically unchanged, suggesting that by the fifth day of stress induction, the animals have acclimatized to the flowerpot technique, i.e., the animals have developed resistance to the stressor.

Significantly decreased serum concentration of testosterone was observed in the stressed group, supporting the observation of Daly *et al*. (2005) and Brownlee *et al*. (2005) that a negative relationship exists between cortisol and testosterone. In earlier years, a direct inhibitory effect of high doses of glucocorticoids upon testicular Leydig cell function in rats has been established, resulting in a decrease in the production of testosterone (Brownlee *et al*., 2005). This occurs via glucocorticoid-induced apoptosis (Hu *et al*., 2008). Brownlee *et al*. (2005) also observed that pharmacologically increased levels of cortisol have a significant negative effect on circulating testosterone. With reduced testosterone concentration in the stressed group, there would be reduced sex drive (libido), and reduced or poor spermatogenesis as testosterone is essential for both.

The gonadotropins were statistically unchanged after stress induction. This finding opposes the observation of Xiao *et al*. (2002), Li *et al*. (2004) and Whirledge & Cidlowski (2010) that stress decreases circulating levels of gonadotropins. This is possibly because of the short stress duration in the present study, as time is required for the effect of stress to reflect on the circulating levels of the hormones. This is confirmed by the significantly reduced serum concentrations of the gonadotropins at 2 weeks after stress-induction, followed by a rise to the same level as the control by the end of the fourth week.

Hormonal levels of testosterone and gonadotropins returned to baseline at the fourth week of rest after stress, around the same period, mating could occur when an estrus female was present. This confirms the importance of testosterone in the initiation of sexual acts (Shulman & Spritzer, 2014).

The results of the study also showed no change in total sperm count following stress. However, the qualities of the sperm cells were altered, specifically, the percentage of normal sperm cells and progressive sperm motility were reduced significantly in the stressed group. These findings are in line with various observations that stress suppresses spermatogenesis resulting in decreased sperm motility and viability (Juárez-Rojas *et al*., 2017; Nargund, 2015). For fertilization to occur after mating, efficient passage of spermatozoa through the cervical mucus is required to meet up with the ovum and this depends on rapid progressive motility (Vogiatzi *et al*., 2022). While some sperm cells may move around the cervix till and outlive their lifespan, only those with the ability to move forward in a straight line can travel through the cervical mucus to fertilize the egg. The high percentage of sperm cells with progressive motility, among other factors, shows an increased probability of conception in the presence of a viable egg (Buck Louis *et al*., 2014).

This study also reported a higher number of headless and tailless sperm cells in the stressed group. Headless sperm cells cannot fertilize the eggs due to the absence of proteolytic enzymes and genetic materials required for fertilization, which are all contained in the head. Likewise, tailless sperm cells cannot fertilize the eggs since they cannot swim to the eggs without their flagella-like tails. Sperm cells with broken midpieces would die off easily since the midpiece houses the mitochondria which is the powerhouse of the cells. Also, a high percentage of head-to-head or tail-to-tail aggregation would result in a lower probability of fertilization since these aggregations would slow down the movement of the sperm cells toward the eggs i.e., impedes progressive motility (Du Plessis *et al*., 2013). An increased percentage of sperm cells with normal morphology shows an increased probability of conception, while a reduced percentage shows a reduced probability of conception (Slama *et al*., 2002).

The alteration in sperm parameters could be attributed to the indirect effect of cortisol on testicular tissues via reduced steroidogenesis, causing changes in Sertoli cells and the blood-testis barrier, and leading to the arrest of spermatogenesis (Nargund, 2015).

The stressed groups showed tubules containing degenerating spermatogenic cells, Sertoli cells, and Leydig cell atrophy as well as mild to severe spermatogenic arrest. The effect of stress on the testes appears to be degeneration of the cellular components including spermatocytes, Sertoli cells, Leydig cells as well as interstitial connective tissue. Spermatogenic arrest as seen in the stressed group is an interruption of sperm cell maturation, which of course impairs the production of mature sperm cells and overtime results in oligospermia (low sperm count) and azoospermia (semen containing no sperm), which are common causes of infertility in males. Since testosterone is one of the most important hormones responsible for both initiation and maintenance of spermatogenesis (Smith & Walker, 2014), a decrease in the serum concentration of testosterone as obtained in this study could account for the spermatogenic arrest observed.

Histopathological changes as shown in the present study clearly demonstrate that acute restraint stress causes marked degeneration of Sertoli cells. These data are in accordance with findings by Rai *et al*. (2003) and Aziz *et al*. (2013). The observed reduction in the serum testosterone of acute restrain stressed rats may be responsible for the disintegration of Sertoli cells. Immobilization stress and heat stress in Wistar rats have been reported to damage Sertoli cells through a decline in androgen (Yazawa *et al*., 1999), oxidative stress (Vallés *et al*., 2014), activation of pro-inflammatory cytokines, and damage of blood-testis-barrier (Xu *et al*., 2015). Nevertheless, the present study reports unchanged oxidative markers by stress, accompanied by increased levels of C-reactive protein.

Histology of the pituitary gland from the group subjected to stress showed disorganized micro architecture comprising a decreased population of acidophils, basophils, and chromophobes. The remaining cells showed variable morphological features, while some appeared normal, others appeared shrunken, and some have pyknotic nuclei. The nuclei of the chromophobes contain inconspicuous nucleoli, which is a sign of a reduction in protein storage. The sinusoids are markedly dilated and congested, taking up spaces originally occupied by parenchymal cells. The glands of the animals subjected to stress have decreased population of neuroendocrine cells, some of which are not viable because of structural derangements. These features give an impression of depletion in protein stores and gonadotropin-producing cells, which will over time reflect as reduced levels of gonadotropins (Ganapathy & Tadi, 2022).

The increased concentration of C-reactive protein observed in the stressed group supports the findings of Brunner *et al*. (1996), Kittel *et al*. (2002), Miller *et al*. (2005), Hamer *et al*. (2006), Nijm *et al*. (2007), Coussons-Read *et al*. (2007) and Ranjit *et al*. (2007). These authors found associations between acute and chronic stress with elevated C-reactive protein and serum fibrinogen concentrations.

Stress is known to activate the sympathetic nervous system in addition to the activation of the HPA axis, resulting in the release of catecholamines and glucocorticoids (Miller & O’Callaghan, 2002). According to Black & Garbutt (2002), the chronic secretion of these hormones may result in endothelial distension and initiate the acute phase inflammatory response involving the release of cytokines and acute phase reactant proteins (C - reactive protein and fibrinogen), though this study observed an unchanged fibrinogen concentration with stress.

Catalase and superoxide dismutase (SOD) activities were unchanged between the stressed and control groups. According to Birben *et al*. (2012) and Sharifi-Rad *et al*. (2018), stressful conditions cause an overwhelming of the body’s antioxidant systems by reactive oxygen species (ROS), causing oxidative stress. Decreased catalase and SOD activity in association with stress has been reported in the past (Zaidi *et al*., 2003; Zafir & Banu, 2009; Priya & Reddy, 2012), but those studies involved chronic stress, unlike the present study which involves acute stress, indicating that the duration of stress determines the stress response.

Significantly reduced pregnancy success rates and litter numbers were observed in the stressed group. There is paucity of data on the effect of paternal stress on pregnancy outcome, though the same has been established for maternal stress (Dunkel Schetter & Tanner, 2012; Traylor *et al*., 2020). The results obtained from this study could be linked to the poor quality of spermatozoa observed. With fewer fertile and viable sperm cells in the stressed group, only a few successful fertilizations could occur, accounting for the reduced pregnancy success rates. Litter weight and litter survival (beyond 1 week) seem to be unaffected by paternal stress, showing that the litter’s development and ability to survive in the external world were like those of the control group. Also, pregnancy outcome after recovery was like that observed in the control group, confirming that complete recovery occurs in males after the stressor is removed and a period of rest is allowed.

The relative expressions of Rhox-5 and Cox-2 genes to the housekeeping gene, glyceraldehyde 3-phosphate dehydrogenase (GADPH) were studied. The significantly reduced expression of the Rhox-5 gene (*p*<0.05) observed in the stressed group could be linked to the poor quality of spermatogenesis observed since the Rhox-5 gene facilitates spermatogenesis (Maclean *et al*., 2005; Hu *et al*., 2010; Welborn *et al*., 2015). There are earlier reports of rodents lacking the Rhox-5 gene being sub-fertile, exhibiting increased germ-cell apoptosis and defects in sperm motility (Maclean *et al*., 2005; Hu *et al*., 2007). This reduced expression of the Rhox-5 gene can be linked to the reduced pregnancy success rate and litter numbers observed in the groups with the stressed males.

Cox-2 gene is a down-regulator of testosterone secretion, and its expression is inversely related to the concentration of testosterone (Wang *et al*., 2005). An increased expression of the Cox-2 gene would lead to a decreased secretion of testosterone and vice versa. The results show no significant difference in the relative expression of the Cox-2 gene between the control and stressed groups. This signifies that the reduced concentration of testosterone observed earlier in the stressed males was not due to down-regulation at the molecular level, but instead because of the atrophic Leydig cells observed in the histology of the testes following stress.
